# A Survey of Public Opinion on Community Cats’ General Health and Relationship Quality with Residents in Urban China

**DOI:** 10.3390/ani14030525

**Published:** 2024-02-05

**Authors:** Xuan Gu, Zilin Zhang, Guo Peng, Anru Ni, Bo Wang, Xiufan Xiong, Yujie Liu, Li Wang

**Affiliations:** 1 Department of Social Work, School of Philosophy and Social Development, Shandong University, Jinan 250100, China; nielsen@sdu.edu.cn (A.N.); emilyxiong1874@outlook.com (X.X.); yujie13627960491@outlook.com (Y.L.); echo19960112@gmail.com (L.W.); 2 Center for Animal Protection Studies, Shandong University, Jinan 250100, China; zilin1126@outlook.com (Z.Z.); guopeng@sdu.edu.cn (G.P.); wangbojqk@gmail.com (B.W.); 3 Department of Philosophy, School of Philosophy and Social Development, Shandong University, Jinan 250100, China; 4 Department of Applied Psychology, Guangdong Peizheng College, Guangzhou 510832, China

**Keywords:** animal welfare, community cat, China, free-roaming cats, human-animal relationship, stray cat, unowned cat, urban ecology

## Abstract

**Simple Summary:**

This study addresses the concern of managing and coexisting with community cats in urban areas. The study was conducted in urban China through a survey involving 5382 participants. It explores the residents’ perceptions of the general health of community cats and the relationships between humans and these feline inhabitants. The findings show that about 70% of participants considered all or most of the community cats to be in good health, and about 60% reported harmonious or non-conflict coexistence. It was found that residents’ relationships with community cats were associated with socio-demographic factors such as education, marital status, and income level. The study highlights the importance of customized approaches to cat management in various cities and communities. This provides useful information for stakeholders to create efficient policies and interventions for the welfare of the community cats and the people who live alongside them.

**Abstract:**

The management and coexistence of community cats in urban areas is a growing concern amid global urbanization. Through a survey-based investigation, we examine the residents’ perceptions of the general health of community cats and human-cat relationships in urban China. The data from 5382 participants revealed that approximately 70% of participants perceived community cats as being in good health, and 60% reported harmonious or non-conflict coexistence between residents and these cats. Around 45% of the participants rescued or helped community cats, 38% expressed their intention to adopt, and 18% complained about the issues of community cats to management staff. Linear, logistic, and multilevel-logistic regressions were employed to examine the associations between the types of cities and communities or the participants’ socio-demographics and the perceived well-being of community cats or human-cat relationships. The results show that the cats in fourth-tier cities (e.g., county-level cities) had poorer living conditions than in first-tier cities (e.g., Beijing), while the cats in urban village communities (e.g., villages in the city) were less likely to exhibit good health than in ordinary commercial housing communities. The results also show that socio-demographic variables, such as educational attainment, marital status, and income level, predicted participants’ relationships with community cats. This study is the first of its kind. It provides valuable insights for stakeholders to develop effective policies and interventions on cat management, emphasizing the need for tailored strategies in diverse urban settings and populations.

## 1. Introduction

Domestic cats (*Felis catus*) are prevalent companion animals worldwide [1,2,3], playing a pivotal role in human lives as they coexist with humans in diverse settings, including families, animal shelters, and communities [4,5,6]. Each domestic cat occupies a place on a spectrum of behavior and lifestyle, ranging from completely independent-living individuals with minimal or no direct human contact to those residing in homes relying on human care [7]. Community cats are positioned in the intermediate range of this spectrum for *Felis catus*. In addition to the term “community cats”, the domesticated cats that are unowned and free-roaming are also referred to as “stray cats” [8,9], “free-roaming cats” [6,10], “feral cats” [11,12], or “outdoor cats” [13,14]. The term “community cats” is widely employed, irrespective of their sociability, gaining popularity as a replacement for the terms stray cats or abandoned cats. This shift acknowledges that the cats are a commensal species highly valued by many residents [15]. Community cats often denote solitary individuals or in a group, maintaining some degree of direct human contact and tolerance, and are provided with food and care to some extent [7]. In this study, we chose to use the term “community cats” (社区猫) to denote unowned and free-roaming cats.

A contentious matter revolves around the terminology that best refers to community cats. When designating them as feral cats or free-roaming cats, researchers often assume a wildlife perspective and adopt strategies from wildlife management or conservation conflict approach [16]. Conservation conflicts occur because the ecological needs and behaviors of wildlife negatively affect the goals of humans or when the goals of humans negatively affect wildlife [17]. In this approach, community cats are regarded as an urgent and pressing ecological threat [12,18,19]. However, some scholars have challenged the findings about the impacts of community cats on wildlife and biodiversity decline. They discussed boundary conditions, considered the role of community cats in rodent control, and noted that anthropogenic activities pose a greater threat to biodiversity than feline behaviors [20,21,22]. Moreover, categorizing animals in urban settings into a binary classification of wild or domesticated oversimplifies the complex realities many city-dwelling species face. According to the definitions by Donaldson and Kymlicka [23], wild animals refer to “those living relatively free of direct human management and meeting their own needs for food, shelter, and social structure” (p. 156), and domesticated animals are “created by human labor to meet specific requirements or whims and are adapted to the conditions of continuous care and solicitude people maintain for them” (p. 74). Rats, for instance, are considered wild animals, yet they are non-domesticated creatures that have adapted to life among humans. As we discussed here, whether the community cats in cities are domestic or wild animals may be viewed differently by different residents or researchers. The domestic/wild dichotomy ignores the vast numbers of wild animals living amongst us, even in the city’s heart. Donaldson and Kymlicka [23], in their work *Zoopolis*, refer to these urban and peri-urban animals as “liminal” animals, that is wild animals living in close association with humans, which signifies their status as neither wilderness nor domesticated animals. Considering the domestication process of cats and their longstanding interdependence with humans [24], we propose a paradigm shift towards an urban ecology perspective instead of a wildlife perspective when investigating the roles and functions of community cats in cities. Urban ecology, conceptualizing cities as social-ecological systems, represents an applied and transdisciplinary field addressing the social, economic, and environmental dimensions of sustainable development [25]. Therefore, urban ecology considers the ecological niche of community cats and explores the natural and humanistic ecology of interactions among humans, other animals, and the natural environment [21,22].

In China, the relationship between humans and cats is distinctive, which is recorded in ancient accounts and cat domestication history. Archaeological research suggests that approximately 5300 years ago, cats were drawn to the ancient Chinese village of Quanhucun by small animals, such as rodents, thriving on the grain cultivated, consumed, and stored by farmers [26]. The study indicates a commensal relationship between humans and cats. Although these cats were not fully domesticated, the evidence affirms their proximity to farmers and the mutually beneficial nature of the association. In contrast to dogs, horses, and cattle, the reliance of cats on humans was limited as recorded in ancient Chinese literature. For example, the Li Ji (also known as Book of Rites) is a core text of the Confucian canon and articulates this sentiment: “The ancient wise men had appointed all these representatives, and it was felt necessary to make this return to them. They met the (representatives of the) cats because they devoured the rats and mice (which injured the fruits) of the fields and the (representatives of the) tigers because they devoured the (wild) boars (which destroyed them). They met them and made offerings to them”. (The Chinese text is based on a translation by Donald Sturgeon in Chinese Text Project: A dynamic digital library of premodern Chinese, Digital Scholarship in the Humanities 2019). (古之君子, 使之必报之。迎猫, 为其食田鼠也。迎虎, 为其食田豕也, 迎而祭之也。) As the poet Liu Zongyuan (773–819) conveyed, “Cats and tigers are worshipped, dogs and horses are buried under drapery”. (猫虎获迎祭, 犬马有盖帷。) In summary, due to the needs of agricultural society, a mutually beneficial relationship developed between humans and cats in China, yet cats exhibited both independence and dependency.

From the 1950s to the 1970s in China, keeping companion animals in urban areas was neither encouraged nor permitted [27]. Companion animals at that time were mainly confined to rural settings, serving utilitarian functions such as cats for rodent control and dogs for home guarding. These rural companion animals had owners but existed in a free-roaming state. Since the 1980s, dog management regulations emerged in Chinese cities such as Beijing. However, there are no regulations for cats or other companion animals in China. Due to the increasing population density and a shift towards vertical living in high-rise structures during urbanization and industrialization after 1979, urban-dwelling cats and dogs as companion animals became predominantly indoor-kept, and most cities in China have fewer outdoor activity space for companion animals [28]. Therefore, there is minimal overlap between household and community cats in Chinese cities. The household status of community cats in Chinese and Western countries is noteworthy and requires comparative investigation.

Returning to contemporary China, cats were the most popular companion animals among urban Chinese residents in 2022, with approximately 65.4 million cats in urban households, surpassing the 51.2 million dogs [29]. However, the dark side of China’s companion animal boom lies in the vast number of cats abandoned by individuals or breeding companies [30,31], contributing to a staggering increase in the community cat population. On average, China witnesses an annual growth of approximately 40 million stray cats [32]. The term “stray cats” (流浪猫) became popular in Chinese discourse to denote unowned cats residing in urban communities [9]. This nomenclature underscores societal concern for these cats’ homeless and abandoned status, whose needs are directly or indirectly addressed by humans. In response to the low welfare of community cats, spontaneous rescuing and helping efforts arise in urban areas, such as community residents’ self-governing organizations dedicated to managing cats and dogs [33]. Meanwhile, people participate in the illicit black market of cat theft and meat trade [34]. Against the above background, it is necessary to investigate the community cats’ survival situations in urban China and their relationship quality with residents. The answers to these questions will facilitate the formulation of regulations on community cats and improve the welfare of community cats and residents. Regrettably, scientific research exploring the interactions between humans and community cats and the urban ecology shaped by this interaction in contemporary Chinese cities is notably limited.

Three dimensions of urban living hold significant ecological importance: (a) Human needs, social patterns, and economics, (b) domestic animals, and (c) pests and public health [35]. (Forman [35] defined pests as organisms that annoy humans, including fungi, bacteria, invertebrate pests (e.g., cockroaches, mosquitoes, ticks), and vertebrate pests (e.g., raccoons and coyotes in the USA). For the vertebrate pests, Forman particularly noted that all of the species are much appreciated by urban residents and also play important positive ecological roles in urban habitats or urban regions as a whole.) The studies on community cats and their relationships with community residents in urban areas constitute an increasingly important intersection of the first and second dimensions. Taking an urban ecology perspective, we initiated a social survey study to investigate the present situation of community cats in urban communities in China. Specifically, our investigation focuses on community cats’ living and health conditions, community residents’ relationships with the cats, and their attitudes towards these felines and relevant policies. Addressing and elucidating these questions contributes to understanding the situations and values of community cats in Chinese cities. Furthermore, the insights will guide practices of cat care and management, legislation, and scientific researches that aim to enhance these feline inhabitants’ welfare.

The data presented is part of the “Social Research Project on Community Unowned Cats in Urban China”, conducted between mid-November 2021 and early February 2022. In the project, we developed a questionnaire to investigate two main issues: (a) The perceived health of community cats and the quality of human-cat relationships in the communities, and (b) the public attitudes towards the community cats and the way of managing the cats (e.g., trapping and killing the cats). The current study focuses on the first issue and explores three research questions. First, what is the general health status of community cats in urban areas? Second, what is the relationship between community cats and residents in urban areas? Third, how do the types of cities and communities and residents’ socio-demographic information predict community cats’ health and the human-cat relationships?

## 2. Materials and Methods

### 2.1. Sampling and Data Collection Procedure

We used a stratified sampling method and defined three strata: Provinces/regions, cities, and urban communities. Firstly, based on economic development and geographical representation, we selected Beijing City (China’s capital and national center), Zhejiang Province, and Shandong Province in the first strata. Secondly, the cities within the provinces or regions were classified into different city tiers. The city tier system is a comprehensive indicator used to measure the socio-economic development level and future potential of different cities. It has been widely applied to urban and regional studies in China [36,37,38,39]. Cities in mainland China are classified into different tiers based on their performance in business, transportation, urbanism, lifestyle diversity, and development potential, using big data and user behavior data [39]. In this study, the type of city is set as a nominal variable with four tiers of cities. The first-, second-, third-, and fourth-tier cities in our sample varied in GDP and population size, as shown in Table 1. Beijing was chosen as the representative of the first-tier cities. Our sample included five districts in Beijing: Chaoyang, Haidian, Xicheng, Fengtai, and Tongzhou. The second-tier cities included the provincial capitals, Jinan and Hangzhou in the current study, which are the capital cities of Zhejiang Province and Shandong Province, respectively. The third-tier cities were prefecture-level cities (地级市) in the two provinces, and the fourth-tier cities were county-level cities or counties (县级市或县城).

Thirdly, the number of sampled communities was determined based on the city tier and the population size of a community. The “community” (*shequ* 社区) is designated as the basic unit of social, political, and administrative organization in urban China [41]. The community in China can be understood in three dimensions: It is a very specific form of a grassroots organization run by a team of officials; It has a distinct territory; It is a social collective formed by people who reside within a defined and bounded district [41,42]. Local investigators were recruited and they were required to obtain permission from their communities’ property management or committee for conducting the investigation before signing up. Based on all of the potentially available communities provided by local investigators, we excluded military residential areas or small communities with insufficient population and determined the communities for the final survey.

The investigators received training before distributing and collecting questionnaires. They also joined supervision provided by the project team to ensure the sampling complied with scientific standards. They were required to confirm participants as community residents, avoid distributing questionnaires to people with specific preferences (such as cat lovers), and prohibit proxy or guided responses. Investigators who completed the investigation received compensation and a certificate of practice.

A total of 150 questionnaires were distributed for large communities with over 5000 residents and 100 questionnaires were distributed for medium-sized communities with 1000 to 5000 residents. The investigators distributed the questionnaires both online and offline. An online questionnaire was set up on the Wenjuanxing platform, a survey service provider in China (also known as Sojump), and distributed through WeChat groups of residents in a community. The investigators also set up booths offline or intercepted people on the roadside. Participants could get access to the online questionnaire by scanning a QR code. For participants who may not be proficient in using smartphones or are reluctant to complete online questionnaires (such as older adults), investigators provided an alternative paper-based questionnaire. Each community had a unique questionnaire link to facilitate tracking the data collection status and each smartphone was limited to one participant’s response. We asked each participant to enter a unique password to fill out a questionnaire, but we did not obtain any identifiable information and it was an anonymous survey.

After completing the online questionnaire, participants received Red Packets by WeChat Pay, in which they got a random amount with an average of CNY 10. Those who completed the paper-based questionnaire received gifts worth approximately CNY 10.

### 2.2. Participants

Our study followed the Declaration of Helsinki and the ethical guidelines for research involving human participants. Prior to answering the questionnaire, all participants provided their informed consent. They were informed that their participation was voluntary and that their privacy would be protected. According to Chinese national policy, the current study did not require ethical approval because it used a survey method and did not collect and report scientific research information on people involved in life sciences and medical issues.

Table 1 presents the number of sampled cities, urban communities, and valid participants. Of the 5728 participants who responded to the questionnaire, we excluded participants who met one of the following criteria: (a) Spent a short time on completing the questionnaire, i.e., less than 120 s; (b) were under the age of 18 or over the age of 86; (c) provided inconsistent information, e.g., retired but with a young age, having children or married under the legal age; (d) selected “other” for gender (N = 12). Ultimately, there were 5382 valid participants available for data analyses.

### 2.3. Measures

We developed a Chinese questionnaire to assess the perceived health status of community cats and the relationship between community cats and participants. We conducted pilot tests to evaluate and improve the understandability of questionnaire items and the smoothness of the data collection process before developing the formal questionnaire and collecting data. We also collected participants’ socio-demographic information. The questionnaire items reported in the current study can be found in the Appendix A.

#### 2.3.1. Community Variables

The community variables included city type and community type. Participants reported their types of community, but the final type was decided by the researchers who interviewed the investigators in each community about the characteristics of the community and housing.

#### 2.3.2. Socio-Demographic Variables

Participants’ socio-demographic information, such as gender, education, age, employment, personal income in the last year (i.e., 2020), and marital status, were collected. To use the nominal variables as predictors in regression models, we converted them into dummy variables. A variable with N categories was converted into N − 1 dummy variables, with the most frequently occurring value of the variable representing 0 and the other value of the variable 1.

#### 2.3.3. Perceived General Health of the Community Cats

Participants were asked how frequently they saw community cats in the community. If they selected “often (more than three days per week)” or “occasionally”, they then proceeded to report where they saw most of the cats appearing in the community, how the community cats got food, the cats’ health condition, and their living condition.

The perceived general health of the community cats was measured by two indicators, i.e., the cats’ health condition and living condition as reported by residents. The living condition was measured using a 5-point Likert scale with 1 = extremely well and 5 = extremely bad. To gauge the health condition of cats, we recoded the responses to the cats’ health condition into a binary outcome variable: 1, a group named good health (N = 3258), for options “All are in good health” and “Most of them are in good health, and a few are sick or disabled”; and 0, another group named bad health (N = 241), for options “Most of them are sick or disabled, and a few are in good health” and “All are sick or disabled”. The response “I don’t notice” was recorded as missing data.

#### 2.3.4. Perceived General Relationship between Residents and Community Cats

Participants rated the general relationship between residents and community cats by answering, “What do you think is the current situation of community residents getting along with unowned cats in the community?” with seven potential responses. We then recoded these responses into a binary outcome variable: 1, a group named coexistence (N = 3215), for options “very harmonious” and “basically coexistent”; and 0, a group named conflict (N = 1332), for options “A small number of residents hate unowned cats in the community”, “Residents generally demand the removal of unowned cats from the community”, and “Property and other managements remove unowned cats from the community”. The responses “I don’t know” and “other situations” were recoded as missing data.

#### 2.3.5. Personal Relationship with Community Cats

There were three indicators to measure the relationship between participants and community cats. Our first indicator of the personal relationship quality was rescuing behavior, which asks whether participants rescued or helped community cats (including feeding and remote sponsorship). Our second indicator of the relationship quality was adopting intention, asking whether participants considered adopting a community cat if they could. Our third indicator of the personal relationship quality was the complaint, which asks whether participants complained to management staff about the issues of community cats. Participants’ responses to the above three questions were coded similarly with “Yes” as 1 and “No” as 0.

### 2.4. Statistical Analyses

Descriptive statistics were employed to summarize the variables. Linear, logistic, and multilevel-logistic regressions were conducted to examine the associations between community or socio-demographic variables and the perceived health of cats or the human-cat relationship. As the living condition of community cats reported in a 5-point Likert scale was a continuous variable, we employed linear regression analysis to investigate the associations between community variables and community cats’ living conditions. As the health condition of community cats and the general relationship between residents and community cats were nominal variables and converted to binary variables, we performed logistic regression analyses to investigate the associations between community variables and community cats’ health condition or general relationship with residents.

Given that the indicators of participant’s relationship with community cats were binary and that participants nested within communities, we fitted multilevel logistic regression models when investigating the extent to which socio-demographic variables and community variables may predict personal relationship with community cats. Multilevel regression modeling can accommodate nested data and include group characteristics in statistical models, which improves the estimates of the effects within groups [43,44]. The variables we analyzed were at two levels. Socio-demographic characteristics of participants, such as age, gender, marital status, and employment, are at the individual level (i.e., level 1 in the regression models). Community variables, such as community type and city type, are at the community level (i.e., level 2 in the regression models). Level 1 variables were nested in level 2. We chose two levels instead of adding different cities as a separate level because, in multilevel logistic modeling, a minimum of 50 level 1 and 40 level 2 units are necessary to accurately estimate small fixed effects [45].

As some responses (e.g., “I don’t know” and “I don’t notice”) were recoded as missing data, the sample sizes varied when different variables served as the outcomes in the regressions. Specifically, the actual sample size was 4621 when the living condition of community cats was the outcome, 3499 when the health condition of community cats was the outcome, and 4547 when the general relationship between residents and community cats was the outcome. Except for the above situations, the analyses included the data of all 5382 participants.

Statistics were performed in SPSS 27 (IBM, New York, NY, USA). A two-tailed *p*-value of < 0.05 was considered statistically significant. Only significant effects are discussed.

## 3. Results

### 3.1. Descriptive Statistics

Table 2 describes the socio-demographic and community variables of the participants, the community cats’ general health, and the relationship between residents and cats as perceived by participants. Approximately 53% of participants were female, and nearly 47% had received a bachelor’s degree or were undergraduate students. The average age was approximately 41 years old. Most participants (approximately 62%) lived in ordinary commercial housing. Other types of communities were presented in a decreasing order: Urban village community, old town community, luxury housing community, indemnificatory housing community, and campus community.

Participants often saw community cats near garbage piles or feeding sites (accounting for about 45%) and reported that people fed community cats regularly or occasionally (accounting for about 51%). Notably, 29% of the participants reported that no residents fed the cats. Thus, we can infer that community cats rely on humans, to varying extents, to acquire sustenance, which may include discarded human food or kibbles provided by humans. The community cats that could be observed were generally healthy, with about 32% of participants reporting that they were all in good health and about 38% reporting that most were in good health. However, about 6% of participants did report that most or all cats were sick or disabled.

For the relationship between residents and community cats in a community, approximately 60% of participants thought that the residents lived with community cats in a harmonious or non-conflict manner. Nevertheless, approximately 24% thought that there were conflicts between residents and community cats in the community. For personal relationships with community cats, approximately 45% of participants rescued or helped community cats, 38% intended to adopt one if conditions were met, and 18% complained about the cats to management staff at the community.

### 3.2. The Associations between Community Variables and the Cats’ General Health or the Human-Cat Relationship

Table 3 displays the results of linear or logistic regression models. A linear regression was performed to assess how the city type and community type would predict the living conditions of community cats as perceived by participants. The assumptions for the linear regression were met (Durbin-Watson statistic = 1.8, 1.0 < VIFs < 1.6, 0.63 < Tolerance < 0.96). The overall regression model was significant, *F* (8, 4620) = 4.0, *p* < 0.001. The community cats inhabiting the communities of first-tier cities had better living conditions than those inhabiting the communities of fourth-tier cities (M = 3.4, SD = 0.86; M = 3.2, SD = 0.98, respectively), *p* < 0.001.

A logistic regression was performed to assess how the city type and community type would predict the likelihood of good health condition of community cats. The overall logistic model was not significant, χ^2^(8) = 13, *p* = 0.10. The effect of urban villages was significant, *p* = 0.04, with the community cats inhabiting urban villages being 0.69 times less likely to have a good health condition than those inhabiting ordinary commercial housing communities.

A logistic regression was performed to assess how the city type and community type would predict the likelihood of a coexisting relationship between residents and community cats in a community. The overall logistic model was significant, χ^2^(8) = 22, *p* = 0.004. Participants in second-tier cities were 1.3 times more likely to have a coexisting relationship than those in fourth-tier cities. Participants in campus communities were 2.1 times more likely to have a coexisting relationship than those in ordinary commercial housing communities.

### 3.3. The Associations between Socio-Demographic and Community Variables and Personal Relationships with Community Cats

Table 4 displays the results from the estimation of multilevel logistic regression models. The three indicators of personal relationship with community cats (i.e., rescuing behavior, adopting intention, and complaint) were the outcome variables separately in each model. The level 1 variables (i.e., participants’ socio-demographic variables) and level 2 variables (i.e., city type and community type) were predictors.

The results of Model 1 showed that the overall regression model was significant, *F* (24, 5357) = 4.5, *p* < 0.001. Compared to participants with a bachelor’s degree and undergraduate students, those with an elementary school degree, a junior high-school degree, or a master’s degree were 0.71, 0.78, or 0.75 times less likely to rescue or help community cats, respectively. Retired participants were 0.77 times less likely to conduct rescuing or helping behaviors than employed participants. Unmarried participants were 1.27 times more likely than married participants to rescue or help the cats. In addition, participants residing in second-tier or third-tier cities were 0.59 or 0.62 times less likely to rescue or help the cats than their counterparts residing in fourth-tier cities, respectively.

The results of Model 2 showed that the overall regression model was significant, *F* (24, 5357) = 8.9, *p* < 0.001. Older adults were more likely to have an adopting intention than younger adults. Unmarried participants were 1.67 times more likely to have an adopting intention than married participants. Participants residing in first-tier or second-tier cities were 0.64 or 0.54 times less likely to have an adopting intention than their counterparts residing in fourth-tier cities. Participants living in the old town communities were 1.68 times more likely than participants living in ordinary commercial housing communities regarding their intentions to adopt community cats.

The results of Model 3 showed that the overall regression model was significant, *F* (24, 5357) = 2.9, *p* < 0.001. Participants with a junior high-school degree were 1.29 times more likely to complain about the issues of community cats than those with a bachelor’s degree and undergraduate students. Older adults were more likely to complain about the issues of community cats than younger adults. Participants whose personal income ranged from 50,000 to 100,000 or from 100,000 to 200,000 were 0.77 or 0.71 times less likely to complain about the issues of community cats than the poorer participants whose personal income was lower than 50,000. Divorced or widowed participants were 1.61 times more likely to complain about the issues of community cats than married participants. In addition, participants from first-tier cities were 0.57 times less likely than those from fourth-tier cities to complain about the issues of the cats. Participants living in old town communities were 1.74 times more likely to complain about the issues of the cats than participants living in ordinary commercial housing communities.

**Figure 1:**
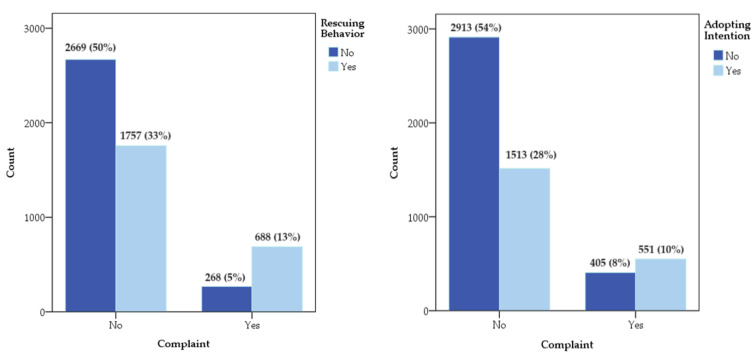
The number and corresponding percentage of options for human-cat relationships.

Figure 1 presents the numbers and corresponding percentages of participants’ responses to assessments regarding personal relationships with community cats. Among the participants who complained about the issues of community cats, which comprised 18% of the sample, 13% reported rescuing or helping the cats, and 10% expressed an intention to adopt a community cat if certain conditions were met. The “issues of community cats” may be understood in two ways. It is possible that participants who filed complaints but rescued or intended to adopt community cats were dissatisfied with the poor or inappropriate management practices related to the care and welfare of the cats. On the other hand, participants who filed complaints and did not care about the community cats may be concerned about issues such as security, public health, or mating calls caused by the cats.

## 4. Discussion

The main aim of the current study was to investigate the public opinion on the general health of community cats and the quality of human-cat relationships in urban communities in China. Our survey revealed that 70% of participants reported that all or most community cats were healthy. And 60% perceived a harmonious or non-conflict coexistence between residents and community cats. Moreover, 45% of participants indicated having rescued or helped community cats. The findings support our proposition to use “community cats” as a substitute for the term “feral cats” or “stray cats”, particularly in Chinese cities. The findings suggest a high level of awareness and concern among residents for the cats residing in their communities, and most of the cats enjoyed a relatively healthy state and coexistence with residents. These findings offer preliminary support for our conceptualization of an urban ecological research framework for studies on community cats. They suggest that urban animals, exemplified by community cats, fall outside the conventional research categories of wild or domestic animals.

Given the absence of city-level, provincial-level, or national-level laws or regulations governing the management of household cats and community cats in China, our findings offer valuable insights for stakeholders to formulate pertinent documents and intervention strategies concerning the management of cats. We revealed the effects of city type and community type. First, participants reported that the community cats exhibited poorer health and that there were more frequent conflicts with residents in fourth-tier cities than in other types of cities. The residents in fourth-tier cities also had a higher probability of rescuing or helping behavior than those in second- or third-tier cities. The findings indicate that in fourth-tier cities, there are more conflicts between residents and community cats, or more conflicts among residents regarding community cat issues. Second, compared to ordinary commercial housing communities, urban village communities were associated with community cats having an inferior health condition; and residents in old town communities had a higher intention for cat adoption and an increased frequency of complaints. The findings suggest that interpersonal conflicts might occur among residents because of differing opinions on the presence of community cats. In sum, it is imperative to allocate increased efforts and resources, focusing on less-developed cities, urban village communities, and old town communities within cities. Previous studies have demonstrated evidence-based measures in controlling the population of community cats, improving the welfare of the cats, and preventing zoonotic diseases. They include (a) neutering cats, a practice strongly endorsed by veterinary professional bodies in the United States and United Kingdom [47,48]; (b) environmental and behavioral modifications to address feline problem behaviors [49], in order to improve the probability of community cats being adopted or to reduce the probability of household cats being abandoned; (c) providing educational interventions for residents, policymakers, and practitioners to familiarize them with the natural behaviors of cats and the ethical treatment of animals [50,51]; and (d) disseminating knowledge and skills related to trap-neuter-return (TNR) or unity-neuter-vaccinate-return (TNVR) programs [52,53]. Simultaneously, it is crucial to investigate the experiences and practices of exemplary cities and communities where the welfare of community cats is ensured and harmonious coexistence with humans is maintained. The successful modules implemented in these locales hold the potential for replication in other similar regions across China.

When analyzing socio-demographic variables, we found that the participants who were more likely to complain about the issues of community cats tended to have lower levels of educational achievement and income and were more likely to be divorced or widowed. These participants closely align with those belonging to a vulnerable group. Vulnerability means facing a significant probability of incurring identifiable harm while substantially lacking the ability or means to protect oneself [54]. One possible explanation for this phenomenon is that due to limited socio-economic resources, vulnerable groups are more susceptible to the adverse effects that community cats may bring, for example, noises from mating calls, high cat populations, and the chaos caused by community cats rummaging through garbage piles. Moreover, vulnerable groups tend to reside in communities with relatively poor living conditions or low-quality property management [55], which may exacerbate conflicts between the groups and community cats. Furthermore, vulnerable groups may need help accessing scientifically valid information, making it difficult to obtain effective methods for addressing community cat issues or fostering positive interactions with community cats. The findings echo the One Health framework, which asserts that the health of people, other animals, and their shared environment are interdependent [56]. Therefore, it is crucial to involve vulnerable groups as integral stakeholders during discussions about community cat management. Allocating more resources and support will enable vulnerable groups to address and resolve the issues arising from cat overpopulation and conflicts between humans and felines, ultimately promoting both parties’ welfare and harmonious coexistence.

The study has two limitations. Firstly, it may be beneficial to inquire whether participants engaged in each behavior separately, such as feeding, TNR, sponsorship, or medical assistance when participants report their rescuing or helping behaviors towards community cats. In a few Chinese communities, residents undertake TNR, medical care, and regular feeding of community cats by the guide of community residents’ self-governing organizations [33]. However, residents in more communities may lack the practices of TNR or medical care and merely feed the cats. A study based on social media data revealed instances of irrational feeding practices by Chinese residents, such as offering leftover human food to cats [9]. While this seemingly benevolent feeding behaviors may be construed as caring and rescuing behaviors from the feeder’s perspective, they can lead to feline health issues and interpersonal conflicts due to hygiene concerns. Secondly, the questionnaire could include more comprehensive variables at the individual or community level to better understand the variables associated with the health status of community cats and the human-cat relationship. For example, future research could measure whether residents live with companion animals, their attitudes and empathy towards animals [57,58], and whether there are individuals or groups involving in cat rescue activities at the community level. These variables have the potential to affect the well-being of community cats and the attitudes of residents towards them.

## 5. Conclusions

The current study examines the welfare condition of community cats in urban China and draws three main conclusions. First, the proportion of people complaining about the issues of community cats is relatively small. Instead, more people exhibit positive attitudes, acceptance, and proactive concern towards these animals. This finding contradicts the biased proportion of Chinese media reports portraying the conflicts between humans and community cats [59,60,61]. Second, in fourth-tier cities, urban village communities, and old town communities, specific interventions and attention are necessary for the health status of community cats and the human-cat relationship. Third, people with lower educational attainment or lower income, or those who are divorced or widowed, are more likely to file complaints. The finding highlights that vulnerable populations may be more susceptible to the adverse effects of community cats’ inadequate welfare or management. Therefore, it is crucial to consider the welfare of vulnerable populations and community cats when considering community cat management.

## Figures and Tables

**Table 1:** Number of sampled cities, communities, and participants (N = 5382).

Type of City	Number of Cities	Number of Communities	Number of Valid Participants	GDP, N × 10^8^ CNY ^#^	GDP% of the National GDP	Population, N × 10^4^
First-tier city: Beijing	1	10	764	40,270	3.5	1414
Second-tier cities: Jinan	1	3	392	11,432	0.99	817
Second-tier cities: Hangzhou	1	3	308	18,109	1.58	835
Third-tier cities in Shandong Province	6	10	1106	39,123	3.41	4991
Third-tier cities in Zhejiang Province	4	8	753	30,679	2.67	1994
Fourth-tier cities in Shandong Province	8	9	1179	4721	0.42	606
Fourth-tier cities in Zhejiang Province	5	9	880	4836	0.41	438
Total	26	52	5382			

^#^ The GDP and population size were based on the provincial statistical yearbook of 2021. Source: [40]. The sum GDP and population size of the third-tier or fourth-tier cities were reported.

**Table 2:** Descriptive statistics of variables among participants (N = 5382).

Variables	Categories	N	Percentage (%)	Mean (SD)	China Census 2020, N × 10^6 #^
Type of City	First-tier city	764	14		
	Second-tier cities	700	13		
	Third-tier cities	1859	35		
	Fourth-tier cities	2059	38		
Type of Community	Old town community	749	14		
	Indemnificatory housing community	110	2		
	Ordinary commercial housing community	3322	62		
	Luxury housing community	174	3		
	Urban village community	920	17		
	Campus community	107	2		
Gender	Female	2848	53		223 (50%)
	Male	2534	47		224 (50%)
Education	Elementary school or below	488	9		59 (13%)
	Junior high-school	785	15		152 (33%)
	Secondary or high school	1107	21		101 (22%)
	College or university	2547	47		140 (30%)
	Graduate or beyond	455	8		9 (2%)
Age	18–85	5382		41 (15)	45 (15)
Employment	Full employment	3090	57		
	Part-time employment	276	5		
	Household work	383	7		
	Full-time students	649	12		
	Unemployment	187	3		
	Retirement	797	15		
Personal Income in the Last Year	<¥50,000	2889	54		
	¥50,000–¥100,000	1526	28		
	¥100,000–¥200,000	674	13		
	>¥200,000	293	5		
Marital Status	Unmarried	1401	26		10.5 (22%)
	Married	3778	70		33.4 (71%)
	Divorced or widowed	203	4		3.3 (7%)
Frequency of Seeing Community Cats	Often	1617	30		
	Occasionally	3004	56		
	Never	289	5		
	I don’t notice.	472	9		
The Living Condition of Community Cats (N = 4621)	5-point Likert Scale	4621		3.3 (0.96)	
The Location Where Community Cats Were Mostly Seen (N = 4621)	Garbage heaps	1461	32		
	Feeding points	619	13		
	Roadside	2026	44		
	Other places	123	3		
	I don’t notice.	392	8		
The Way How Community Cats Got Food (N = 4621)	Some people feed them regularly.	639	14		
	Some people feed them occasionally.	1765	38		
	No one feeds them and they find food themselves.	1323	29		
	I don’t notice.	894	19		
The Health Condition of Community Cats (N = 4621)	All are in good health.	1492	32		
	Most of them are in good health; A few are sick or disabled.	1766	38		
	Most of them are sick or disabled; A few are in good health.	219	5		
	All are sick or disabled.	22	0.5		
	I don’t notice.	1122	24		
Perceived General Relationship between Residents and Community Cats	Very harmonious.	818	15		
	Basically coexistent.	2397	45		
	A small number of residents hate unowned cats in the community.	1083	20		
	Residents generally demand removal of unowned cats from the community.	173	3		
	The management staff remove unowned cats from the community.	76	1		
	I don’t know.	816	15		
	Other situations.	19	0.4		
Personal Rescuing or Helping Behavior	Yes	2445	45		
	No	2937	55		
Personal Adopting Intention	Yes	2064	38		
	No	3318	62		
Personal Complaint	Yes	956	18		
	No	4426	82		

^#^ Based on the seventh national census in 2020 in China, we calculated the statistics data of the urban population. Due to the availability of the census data, the age range of the population is 18–84 years old when the statistics data of education were calculated and the age range is 20–84 years old when the statistics data of gender and age were calculated. The blank space indicates no data are available. Source: [46].

**Table 3:** Results of a linear model and two logistic regression models examining the effects of community variables on the general health of the community cats and the quality of human-cat relationship in urban China.

		Living Condition of Community Cats	Health Condition of Community Cats	General Relationship between Residents and Community Cats
		N = 4621	N = 3499	N = 4547
**Community Variables**		*β* [95%CI]	OR [95%CI]	OR [95%CI]
Type of City	Fourth-tier cities (ref.)			
	First-tier city	0.06 ** [0.07, 0.27]	1.12 [0.70, 1.80]	1.17 [0.92, 1.48]
	Second-tier cities	−0.02 [−0.14, 0.05]	0.90 [0.57, 1.41]	1.29 * [1.03, 1.61]
	Third-tier cities	−0.02 [−0.12, 0.03]	0.98 [0.70, 1.38]	1.08 [0.91, 1.27]
Type of Community	Ordinary commercial housing community (ref.)			
	Old town community	−0.003 [−0.09, 0.08]	0.98 [0.66, 1.45]	1.20 [0.98, 1.47]
	Indemnificatory housing community	0.03 [−0.01, 0.37]	2.14 [0.66, 6.94]	1.46 [0.92, 2.32]
	Luxury housing community	0.03 [−0.02, 0.30]	1.54 [0.60, 3.91]	0.98 [0.68, 1.42]
	Urban village community	−0.03 [−0.16, 0.01]	0.69 * [0.48, 0.98]	1.13 [0.94, 1.37]
	Campus community	−0.01 [−0.27, 0.16]	4.05 [0.54, 30.58]	2.05 * [1.15, 3.65]

*β* is standardized regression coefficient; OR, Odds Ratio; CI, Confidence Interval. * *p* < 0.05, ** *p* < 0.01.

**Table 4:** Results of multilevel logistic regression models examining the effects of socio-demographic and community variables on personal relationships with community cats in urban China (N = 5382).

		Rescuing Behavior	Adopting Intention	Complaint
		(Model 1)	(Model 2)	(Model 3)
		OR [95%CI]	OR [95%CI]	OR [95%CI]
	Fixed effects			
	Intercept	13.88 * [1.22, 158.21]	4.31 [0.53, 35.27]	15.65 * [1.14, 214.82]
Socio-demographic Variables				
Gender	Female (ref.)			
	Male	0.95 [0.84, 1.06]	0.99 [0.88, 1.12]	1.12 [0.97, 1.31]
Education	College or university (ref.)			
	Elementary school or below	0.71 * [0.54, 0.93]	0.81 [0.61, 1.09]	1.02 [0.71, 1.46]
	Junior high-school	0.78 * [0.63, 0.95]	1.20 [0.98, 1.48]	1.29 * [1.01, 1.65]
	Secondary or high school	0.88 [0.75, 1.03]	1.14 [0.97, 1.34]	1.18 [0.96, 1.44]
	Graduate or beyond	0.75 * [0.59, 0.95]	0.87 [0.68, 1.11]	1.02 [0.74, 1.39]
Age	18–85	1.00 [1.00, 1.01]	1.02 *** [1.01, 1.02]	1.02 ** [1.01, 1.02]
Employment	Full employment (ref.)			
	Part-time employment	1.09 [1.00, 1.01]	1.01 [0.78, 1.32]	0.89 [0.64, 1.24]
	Household work	0.88 [0.69, 1.12]	0.86 [0.67, 1.11]	0.77 [0.56, 1.06]
	Full-time students	1.00 [0.78, 1.28]	0.96 [0.75, 1.23]	0.93 [0.68, 1.27]
	Unemployment	0.80 [0.58, 1.11]	0.81 [0.58 1.12]	1.02 [0.70, 1.49]
	Retirement	0.77 * [0.61, 0.98]	0.91 [0.70, 1.17]	0.77 [0.56, 1.06]
Personal Income in the Last Year	<¥50,000 (ref.)			
	¥50,000–¥100,000	0.98 [0.84, 1.13]	0.89 [0.76, 1.03]	0.77 ** [0.64, 0.92]
	¥100,000–¥200,000	1.09 [0.89, 1.33]	0.96 [0.78, 1.18]	0.71 * [0.55, 0.93]
	>¥200,000	0.87 [0.65, 1.15]	0.97 [0.73, 1.31]	0.70 [0.48, 1.01]
Marital Status	Married (ref.)			
	Unmarried	1.27 * [1.04, 1.54]	1.67 *** [1.37, 2.04]	0.80 [0.63, 1.03]
	Divorced or widowed	1.31 [0.97, 1.78]	0.97 [0.70, 1.35]	1.61 * [1.12, 2.32]
Community Variables				
Type of City	Fourth-tier cities (ref.)			
	First-tier city	0.65 [0.41, 1.02]	0.64 * [0.44, 0.95]	0.57 * [0.35, 0.92]
	Second-tier cities	0.59 * [0.36, 0.98]	0.54 ** [0.36, 0.92]	0.90 [0.55, 1.45]
	Third-tier cities	0.62 * [0.43, 0.89]	0.79 [0.58, 1.06]	0.76 [0.54, 1.09]
Type of Community	Ordinary commercial housing community (ref.)			
	Old town community	1.26 [0.82, 1.94]	1.68 ** [1.18, 2.39]	1.74 ** [1.15, 2.65]
	Indemnificatory housing community	0.59 [0.21, 1.68]	0.70 [0.30, 1.65]	1.23 [0.46, 3.30]
	Luxury housing community	1.05 [0.39, 2.87]	0.70 [0.31, 1.57]	1.41 [0.56, 3.53]
	Urban village community	0.97 [0.64, 1.48]	0.96 [0.68, 1.36]	0.98 [0.66, 1.48]
	Campus community	0.90 [0.30, 2.68]	1.14 [0.47, 2.81]	0.28 [0.07, 1.10]
	Random effects			
	Intercept	0.22 *** [0.13, 0.37]	0.13 ** [0.07. 0.24]	0.17 ** [0.09, 0.32]

OR, Odds Ratio; CI, Confidence Interval; * *p* < 0.05, ** *p* < 0.01, *** *p* < 0.001.

## Data Availability

The data presented in this study are available on reasonable request from the corresponding author.

## Appendix A. The Questionnaire Items Reported in the Study

**1. Which community do you currently live in?** [Single-choice question]

◯ *[The name of the community]* (this community)

◯ Other community within the city

◯ Other community outside of the city

**2. What is your community type?** [Single-choice question]

◯ Old town community

◯ Indemnificatory housing community (e.g., low-rent housing, public rental housing)

◯ Ordinary commercial housing community

◯ Luxury housing community (e.g., villa area, high-class residential area)

◯ Urban villages community (e.g., urban community recently transformed from rural community, “villages in city”)

◯ Other _________________

**3. What is your gender?** [Single-choice question]

◯ Male

◯ Female

◯ Other

**4. What is your age? (Please fill in a whole number, e.g., 26)** [Fill in the blank]

______________________________________________________________

**5. What is your current highest level of education (including those you are currently enrolled in)?** [Single-choice question]

◯ Below elementary school

◯ Elementary school

◯ Junior high school

◯ Secondary or high-school

◯ College or university

◯ Graduate or beyond

**6. What is your current employment?** [Single-choice question]

◯ Full Employment

◯ Part-time job

◯ Household work (refers to staying at home to take care of other family members, commonly known as “housewife” or “househusband”)

◯ Full-time students

◯ Unemployment

◯ Retirement

**7. What was your personal gross income in 2020?** [Single-choice question]

◯ Less than 10,000 CNY

◯ 10,000–20,000 CNY

◯ 20,000–50,000 CNY

◯ 50,000–100,000 CNY

◯ 100,000–200,000 CNY

◯ 200,000 RMB and above

**8. What is your current marital status?** [Single-choice question]

◯ Unmarried

◯ Married

◯ Divorced

◯ Widowed

**9. How frequently do you see community unowned cats (commonly known as “stray cats”) in the community where you live?** [Single-choice questions]

◯ Often (more than three days a week)

◯ Occasionally

◯ Never (Please skip to Question 14)

◯ I don’t notice. (Please skip to Question 14)

**10. Where do you mostly see unowned cats in the community?** [Single-choice questions]

◯ Garbage heaps

◯ Feeding points

◯ Roadside

◯ Others _________________

◯ I don’t notice.

**11. What is the pattern of food seeking of unowned cats in your community?** [Single-choice question]

◯ Some people feed them regularly.

◯ Some people feed them occasionally.

◯ No one feeds them and cats find food their own.

◯ I don’t notice.

**12. What is the health condition of unowned cats in your community?** [Single-choice question]

◯All are in good health.

◯ Most of them are in good health, and a few are sick or disabled.

◯ Most of them are sick or disabled, and a few are in good health.

◯ All of them are sick or disabled.

◯ I don’t notice.

**13. With 5 being extremely good living situations and 1 being extremely poor living situations, how would you rate the living situations of unowned cats in your community?** [Single-choice question]

◯ 1

◯ 2

◯ 3

◯ 4

◯ 5

**14. Would you consider adopting an unowned cat from the community if you had the conditions to do so?** [Single-choice question]

◯ Yes

◯ No

**15. Have you rescued an unowned cat in your community?** (Note: Feeding and remote sponsorship are also considered as rescuing behaviors) [Single-choice question]

◯ Yes

◯ No

**16. Have you complained about the issues of unowned cats to management staff in the community?** [Single-choice question]

◯ Yes

◯ No

**17. What do you think is the current situation of community residents getting along with unowned cats in the community?** [Single-choice question]

◯ Very harmonious

◯ Basically coexisting

◯ A small number of residents hate unowned cats in the community.

◯ Residents generally demand the removal of unowned cats from the community.

◯ Property and other managements remove unowned cats from the community.

◯ I don’t know

◯ Other situations_________________
